# The Effect of Motion Correction on Quantitative Perfusion Indices and Threshold-Based Interpretation in ^13^N-Ammonia Dynamic PET Myocardial Perfusion Imaging

**DOI:** 10.3390/diagnostics16162610

**Published:** 2026-08-17

**Authors:** Ajay Kumar Chaudhary, Zekun Pang, Fukai Zhao, Jing Ni, Haoran Guo, Yue Chen, Jiao Wang, Jianming Li

**Affiliations:** 1 International Medical School, Tianjin Medical University, Tianjin 300070, China; chyajay112@gmail.com; 2 Department of Nuclear Medicine, Clinical Cardiovascular Institute, Tianjin Medical University, Tianjin 300457, China; kimi0925@126.com (Z.P.); zhao_fukai@163.com (F.Z.); 18200135996@163.com (J.N.); ghaoran_02@163.com (H.G.); cyzhuque@163.com (Y.C.); 18622888846@163.com (J.W.); 3 Department of Nuclear Medicine, TEDA International Cardiovascular Hospital, Tianjin University, Tianjin 300457, China

**Keywords:** positron emission tomography, myocardial perfusion imaging, myocardial blood flow, myocardial flow reserve, motion correction

## Abstract

**Background/Objectives:** The effect of cardiac motion on dynamic positron emission tomography (PET) myocardial perfusion imaging (MPI) could distort the quantitative perfusion indices during acquisition, depending on direction, magnitude, and vascular territory. This study aimed to assess the impact of motion correction (MC) and its influences on quantitative perfusion indices using a comprehensive range of established statistical methods. **Method:** We retrospectively analyzed 171 patients’ data who underwent ^13^N-ammonia dynamic PET/CT-MPI. All the rebinned listmode data were transferred to the dedicated workstation for MC and quantification, yielding paired MC and non-motion correction (NMC) datasets for both phases. Frame-by-frame post-MC vectors were used to characterize motion magnitude and direction. Paired comparisons, directional analysis, association, agreement, accuracy, precision, and clinical reclassification were performed. **Results:** Motion occurred during both acquisition phases, but it was more frequent, more heterogeneous, and generally greater during stress, with the most notable displacement along the Y-axis. MC had a limited impact on the rest myocardial blood flow (MBF) but showed more significant effects on stress MBF, myocardial flow reserve (MFR), and non-invasive fractional flow reserve (niFFR) -like index, especially in the left anterior descending artery (LAD). Although MC and NMC values remained strongly associated for MBF and MFR, they were not fully interchangeable. The bidirectional reclassification of MFR and niFFR-like index showed that MC responded to the direction and magnitude of motion rather than a simple increase the values. **Conclusions:** MC exerts a structured, bidirectional, and motion-dependent influence on quantitative dynamic PET-MPI and has an impact beyond simple numerical adjustment.

## 1. Introduction

Dynamic positron emission tomography (PET) myocardial perfusion imaging (MPI) offers a quantitative measurement of absolute myocardial blood flow (MBF) and myocardial flow reserve (MFR), providing physiologic information that goes beyond relative perfusion assessment alone [1,2]. These indices are increasingly employed to evaluate coronary artery disease (CAD), diffuse atherosclerosis, microvascular dysfunction, and risk assessment, but the clinical utility depends on technically stable acquisition, accurate contour definition, and meticulous quality control throughout the dynamic study [1,2,3].

In dynamic PET-MPI, patient motion of several millimeters to centimeters commonly occurs during rest and stress acquisitions, especially under pharmacologic stress [4,5,6,7]. Cardiac motion during pharmacologic stress is common and can be exacerbated by myocardial creep, respiratory variation, and changing thoracic mechanics, which affect emission data and attenuation alignment, thereby altering the time–activity curve (TAC), kinetic modeling, and subsequent MBF and MFR estimates [4,5,6,7,8,9]. Simulation and clinical studies show that direction and timing of motion can significantly disrupt regional MBF and MFR estimation, with uncorrected motion leading to substantial quantitative errors [4,6,7,10].

To address this, a range of motion correction methods has been developed, from manual or semi-automatic frame-by-frame rigid registration to fully automated three-dimensional algorithms, as well as more advanced techniques integrated into hybrid imaging workflows that modify MBF and MFR estimates, improve repeatability and reproducibility, and affect diagnostic performance [4,5,6,7,8,9,10,11,12]. However, the reported effects of MC vary across studies in terms of dominant motion direction, regional correction patterns, and quantitative changes, suggesting that the impact of MC is likely dependent on each specific dataset and dependent on the particular motion pattern, rather than a simple, uniform shift in perfusion values [4,5,7,13].

An additional consideration is that motion-related perturbations may affect not only MBF and MFR but also lesion-specific physiologic indices derived from PET flow data, such as non-invasive fractional flow reserve (niFFR) or relative flow reserve (RFR). Whereas MFR reflects integrated territory-level flow behavior across epicardial and microvascular disease, an FFR-like index (niFFR) may provide complementary lesion-focused information that could influence interpretation or risk assessment [14,15,16]. In this setting, understanding how MC affects both the territory-level and lesion-specific quantitative measures is therefore relevant to a broader interpretation of dynamic PET MPI.

Therefore, this study aims to characterize the influence of MC on dynamic PET-MPI using a range of established statistical methods. Instead of relying on an external gold standard for physiologic validation, we described frame-wise motion during rest and stress, examined the numerical differences between MC and (non-MC) NMC perfusion indices, and explored whether these patterns were consistent across paired comparisons, directional change, regression, agreement, reclassification, and motion severity. By combining motion analysis with perfusion metrics, we aimed to describe the magnitude and direction of MC-related shifts and to examine their potential influence on threshold-based classifications.

## 2. Materials and Methods

### 2.1. Study Population

**Figure 1:**
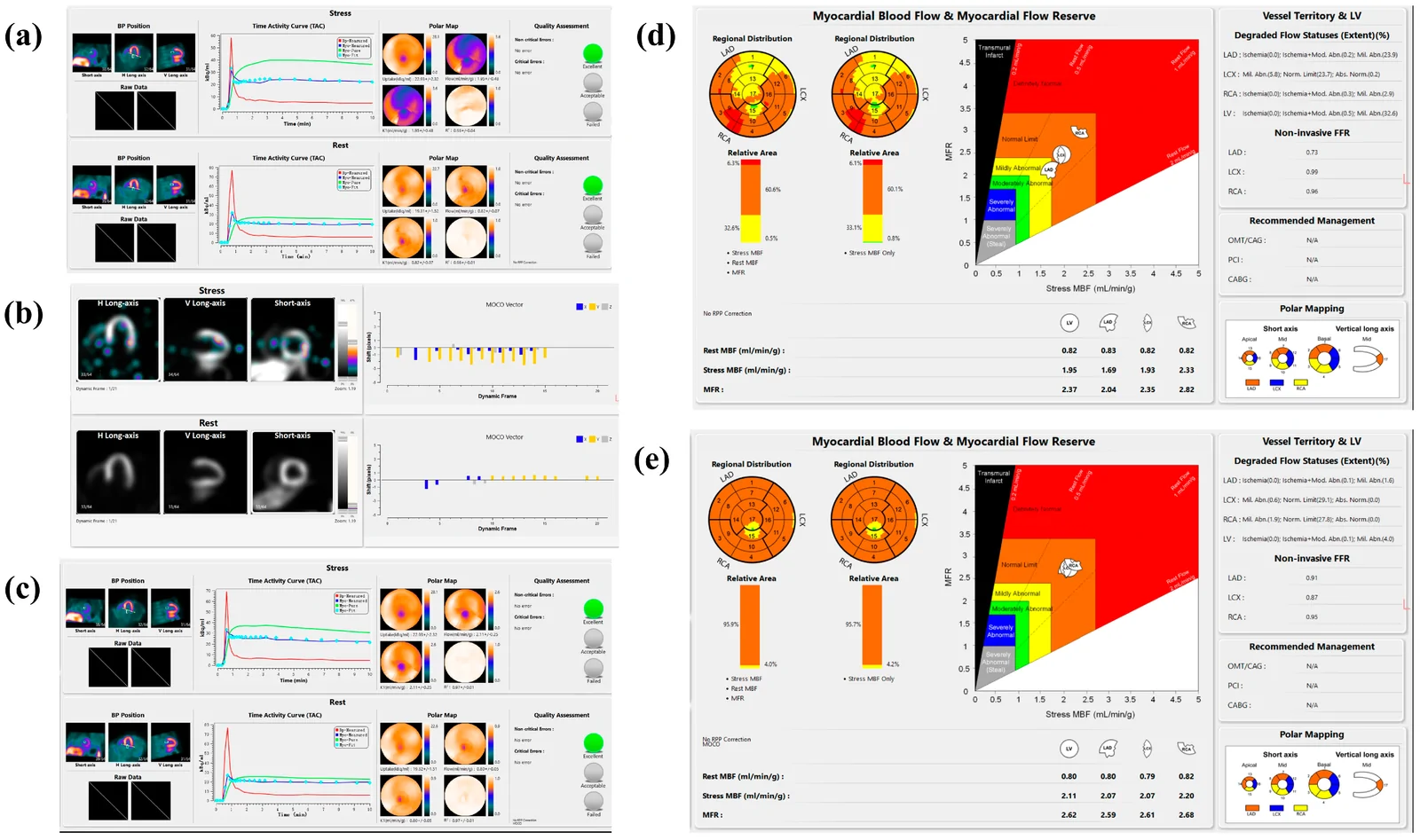
Dynamic PET MPI quantitative analysis before and after MC of a 57-year-old female. Footnote: TAC stress (top) and rest (bottom) before (**a**) and after MC (**c**) display blood pool/LV localization, polar map, and quality assessment; a frame-by-frame post-processing motion graph illustrates displacement across the 3D translational axis (X, Y, Z) along the dynamic frames (**b**); global and regional MBF (rest, stress), MFR, and niFFR-like index for NMC (**d**) and MC (**e**), highlighting the quantitative impact of MC. LV, left ventricle; LAD, left anterior descending artery; LCX, left circumflex artery; RCA, right coronary artery; BP, blood pool; H Long axis, horizontal long axis; V Long axis, vertical long axis; TAC, time–activity curve; MIP, myocardial perfusion imaging; MBF, myocardial perfusion imaging; MFR, myocardial perfusion reserve; MC, motion correction; NMC, non-motion correction.

This study included consecutive patients who underwent dynamic ^13^N-ammonia PET/CT-MPI at TEDA International Cardiovascular Hospital between 10 November 2025, and 31 January 2026, and whose imaging data received an “excellent” rating during automated quality assessment (QA) (i.e., defined as absence of critical/non-critical errors) in both MC and NMC on the MyoFlowQ Workstation (V2.0) (Beijing Larkcloud Biomedical, Beijing, China) during processing (Figure 1a,c). Patients with substandard imaging quality or incomplete data, or discordant QA ratings or distorted TAC were excluded (Supplementary Figure S1). Of the 185 patients, 171 met the predefined QA criteria and were included in the final analysis. Informed consent for clinical imaging and secondary research use of de-identified data was obtained from all participants.

### 2.2. Imaging Equipment, Acquisition Protocols, and Dynamic Image Reconstruction

For PET/CT MBF quantification, a GE Discovery Elite PET/CT scanner (Discovery NM690, GE Healthcare, Waukesha, WI, USA) was used for rest and adenosine-stress dynamic scans. The imaging agent ^13^N-NH_3_·H_2_O was produced by our department’s medical cyclotron (Qilin, GE Healthcare). Patients were required to avoid caffeine and theophylline for 24 h before imaging, discontinue cardiovascular medications when clinically permitted, and refrain from smoking on the day of the procedure.

A one-day rest/adenosine-stress imaging protocol [3] required patients to lie supine and drink 300–500 mL of water beforehand to reduce liver spill-in artifacts [17]. Both phases received the same tracer dose of about 222–370 MBq. Initially, a scout image was acquired for anatomical localization, followed by a low-dose CT scan (120 kV tube voltage, 50 mA tube current) for attenuation correction before PET acquisition in both dynamic phases. The dynamic image acquisition was initiated 10–15 s before a rapid intravenous injection of the tracer bolus via a 3-way tube, followed by a 10 mL saline flush to clear any residual tracer. After injection, each dynamic acquisition lasted for 10 min. After the rest scan, a delay of 30–50 min before the start of stress dynamics. Pharmacologic stress was induced using adenosine infused at 0.14 mg/kg/min for 6 min, with the stress scan starting at 2 min and 50 s after initiation of infusion [1,3]. Gated acquisition lasted 8 min after dynamic acquisition in both phases.

The listmode data were rebinned into dynamic frames (10 × 10 s, 5 × 20 s, 5 × 60 s, and 1 × 100 s). Following this, image reconstruction includes standard physical correction (attenuation, scatter) using VUE Point HD recon, with iterative OSEM reconstruction (24 subsets, 2 iterations), a 128 × 128 matrix, 425–650 keV energy window, and a Hanning filter (12 mm cutoff).

### 2.3. Motion Graph and MBF Quantification

The rebinned listmode dynamic frame data were transferred to the MyoFlowQ workstation (V2.0) for MC and quantification. The vendor-provided MC algorithm performs automated frame-based registration and estimates 3D translational displacement along the X (±left/right), Y (±anterior/posterior), and Z (±superior/inferior) axes, followed by spatial realignment across all dynamic frames. Raw pre-correction motion parameters are not retained; instead, it displays a post-processing motion graph illustrating the applied displacement vectors in pixel units (1 pixel shift = 4.375 mm; isotropic voxel resolution of 4.375 × 4.375 × 4.375 mm) (Figure 1b). The MC process operates on attenuation-corrected PET emission data and does not modify the attenuation map or re-register.

For comparison, the NMC and MC series were generated by disabling or enabling the MC function (Figure 1d,e), respectively. The impact of MC was assessed by comparing paired MBF, MFR, and niFFR-like indices between datasets.

First, rest/stress-phase MPI dynamic rebinned listmode frames were summed over 300–600 s to obtain static images (reference images). These images were paired with respective dynamic series and reoriented into standard cardiac planes for framewise correction of the dynamic frames. The MC algorithm calculated the myocardium’s center of mass (COM) in dynamic and reference images using a 3D volume created from an ellipsoid model that automatically defined the myocardial centerline. The mitral valve plane was manually adjusted using the final dynamic frame, with manual refinement as needed. The COM of each dynamic frame was then compared with that of the reference images to generate 3D translational vectors. In early dynamic frames, where radioactivity stays in the blood pool, image intensities were inverted to enhance the myocardium for COM calculation. For intermediate frames with radioactivity distributed between the blood pool and the myocardium, COM was not calculated, and vectors were set to null. Translational vectors for early and late frames were calculated and applied to register dynamic images with the reference image, respectively. After registration, the dynamic frame was carefully checked by a nuclear cardiologist for proper registration frame-by-frame and adjusted manually when necessary. Final 3D translational vectors were algorithm-generated by the registration process to present the degree of motion and then treated to correct for motion in dynamic data. Additionally, the algorithm also generated polar maps and extracted TAC for both phases of the study.

Following MC, the arterial TAC was derived using a rectangular volume of interest placed over the left ventricular and arterial cavities (1:2 ratio) with manual refinement (Figure 1a,c). Kinetic analysis was performed using a one-tissue (two-compartment) model to estimate the tracer uptake rate constant (K1), while also incorporating corrections for blood-pool spill-over, partial volume effects, and residual tracer activity [18]. Rest and stress MBF were calculated directly, MFR was calculated as the stress-to-rest MBF ratio, and a niFFR-like index was automatically derived by the software as the ratio of hyperemic MBF in the lowest flow territory to the highest flow contralateral territory on the 17-segment polar map. This approach is consistent with the concept of relative flow reserve (RFR) [16] but presented as an exploratory metric in this study.

## 3. Statistical Analysis

Because motion vectors and perfusion indices were non-normally distributed by the Shapiro–Wilk test, non-parametric tests were used throughout. Continuous variables are reported as medians with interquartile range (IQR: P25–P75%), and categorical variables as counts and percentages. Motion vector data were analyzed at the frame level for descriptive purposes; however, to account for the nested structure of these data (multiple frames per patient and per axis, positive or negative directions), frame-wise non-zero displacement along the X, Y, and Z axes was compared using the Friedman test with Bonferroni correction, and motion was further described by median absolute value, IQR, directional bias, overall motion magnitude, and stratified motion category distribution across indices.

The effect of MC relative to NMC was assessed using the Wilcoxon signed-rank test for paired comparisons, and for all paired comparisons, the Hodges–Lehmann (HL) estimator with its 95% confidence interval (CI) was reported. To control Type-I error across multiple comparisons, Bonferroni–Holm correction was applied to all paired comparisons, with corrected *p*-values reported alongside uncorrected values. Spearman correlation was used for monotonic associations. Passing–Bablock regression was used to assess constant and proportional bias. Agreement was evaluated using Bland–Altman analysis with median bias and percentile-based limits of agreement (LoA), together with Lin’s concordance correlation coefficient (CCC) for accuracy and precision. Categorical reclassification of MFR and niFFR was analyzed using the McNemar test with Spearman’s rho for association. All tests were two-sided, with *p* < 0.05 considered statistically significant. Statistical analyses were performed in R 4.5.2 (R Foundation for Statistical Computing, Vienna, Austria).

## 4. Results

Of the 185 patients screened, 14 were excluded due to balanced ischemia, discordant QA rating, failed QA in either dataset, or distorted TACs, leaving 171 for final analysis (Supplementary Figure S1). The median age was 61 years (IQR: 54–69), with a predominantly male cohort (55.6%) and a median BMI of 25.8 kg/m^2^ (IQR: 23.9–28.5). Most patients were aged ≥60 years (56.14%) and overweight (47.37%) or obese (16.96%). Additional baseline characteristics are summarized in Table 1.

### 4.1. Motion Vector Graph

The MyoFlowQ software (V2.0) provides post-correction motion plots representing frame-to-frame 3D translational displacement vectors along the X, Y, and Z axes in pixel units. As the platform reports only motion estimates applied during correction, raw pre-correction trajectories were unavailable; therefore, motion assessment was limited to descriptive rather than direct comparison.

**Figure 2:**
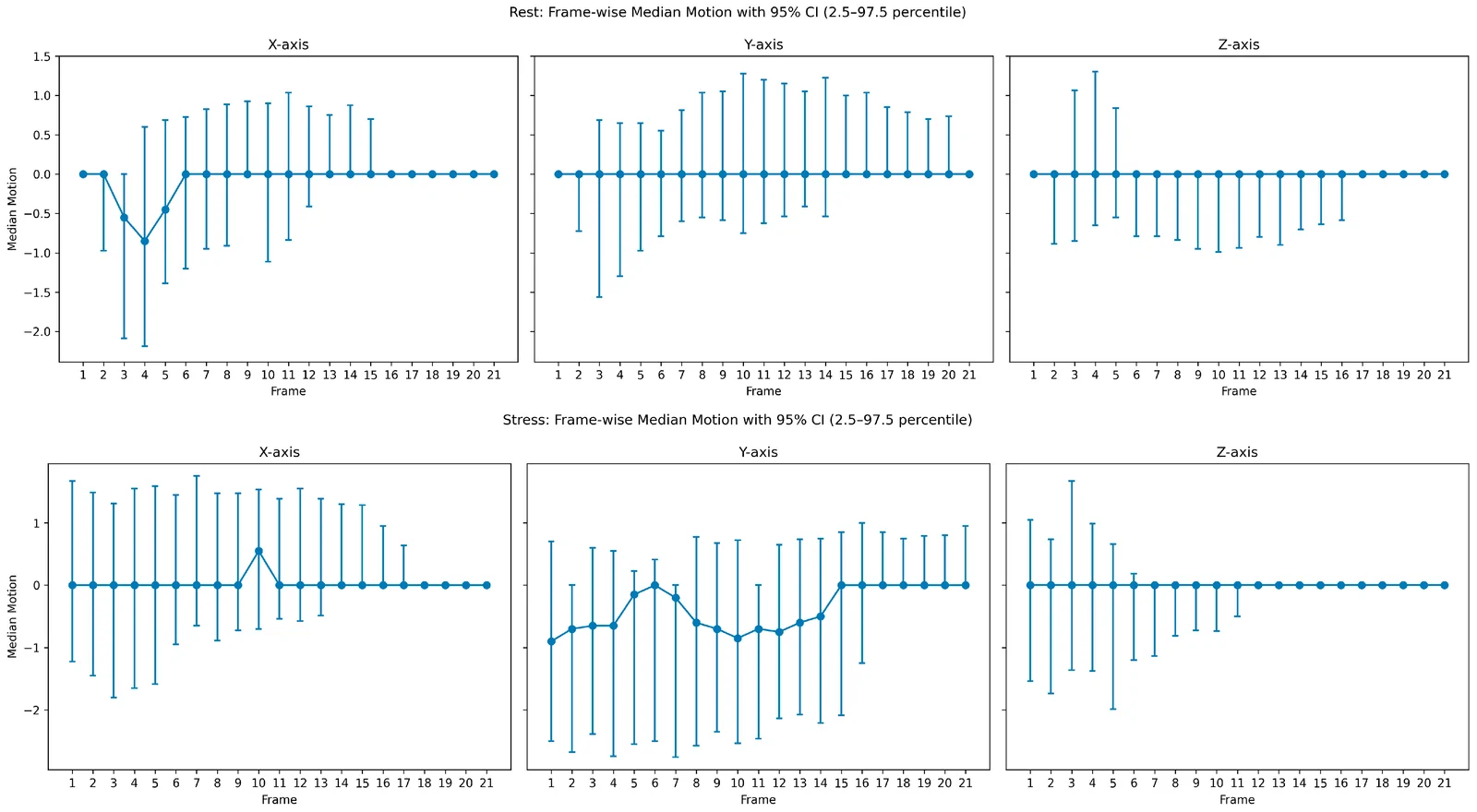
Frame-wise median motion along the motion vectors during the PET dynamic scans. Footnotes: Median displacement (points) with percentile based 95% CI was shown for each of the 21 frames measured in pixel shifts, top (rest) and bottom (stress).

Frame-wise analysis showed non-zero motion detected in every patient along all three axes during both phase acquisitions, but the burden of motion was clearly greater during stress (Figure 2, Table 2). At rest, the median absolute value was 0.75 pixels along the X-axis, 0.7 pixels along the Y-axis, and 0.65 pixels along the Z-axis. During stress, motion increased across all axes to 0.8, 1, and 0.7 pixels, respectively, with the largest increase observed along the Y-axis (Table 2). Directionally, rest motion was prominently right, anterior, and inferior, while stress motion shifted mainly left, posterior, and inferior due to chest wall mechanics caused by the stressor; moreover, there was also motion in the reverse direction too (Figure 2 and Table 2).

This pattern was supported by axis-wise comparison of motion amplitude. The Friedman test showed significant heterogeneity across axes at both phases, but the pattern was more marked during stress. At rest, the overall difference was significant (X^2^ (2) = 11.83, *p* = 0.003), driven mainly by a difference between the X and Z axes. During stress, heterogeneity was more pronounced (X^2^ (2) = 28.13, *p* < 0.001), with all pairwise comparisons remaining significant after Bonferroni correction, corresponding to a more complex and anisotropic motion pattern during stress.

When stratified by magnitude (1 pixel = 4.375 mm), most motion events fall within the >3–7 mm range in both phases; however, >7 mm was distinctively more common during stress, particularly along the Y-axis, where it accounted for 18.15% of displaced frames, compared with only 1.65% at rest (Supplementary Table S1). This provides the mechanistic basis for why any downstream effect of MC would be expected to emerge more strongly in stress-derived perfusion indices.

### 4.2. Impact of MC on Quantitative Perfusion Indices

Consistent with the greater motion burden observed during stress acquisition, MC had a limited effect on resting MBF (rMBF) but a more evident impact on stress MBF (sMBF), MFR, and niFFR-like index across the entire cohort; these comparisons included all subjects and were not limited to those with detectable inter-frame motion (Table 3; Supplementary Table S2 and Figure S2). Although the LV-rMBF median remained numerically unchanged at 0.73 mL/min/g, MC was associated with a small downward paired shift was observed after MC (HL: −0.01 mL/min/g; r = 0.30; Holm-adjusted *p* = 0.008). Regionally, rMBF was lower after MC in LCX and RCA (r = 0.41 and 0.28; Holm-adjusted *p* < 0.001 and 0.009, respectively), whereas LAD did not differ after adjustment. Therefore, the observed rMBF difference after MC was small in absolute magnitude.

In contrast, sMBF was higher after MC in the global LV and in the LAD and LCX territories. The largest difference was observed in the LAD, with a median paired difference of 0.07 mL/min/g (HL: 0.06 [0.04–0.08]; r = 0.56; Holm-adjusted *p* < 0.001). Global LV and LCX showed smaller increases, whereas RCA did not differ after adjustment (Table 3; Supplementary Table S2).

MC was also associated with an increase in global and regional flow-derived indices. MFR was higher after MC in the global LV, LAD, and LCX territories, with the largest difference observed in LAD, 0.12 (HL: 0.08 [0.06–0.11]; r = 0.51; Holm-adjusted *p* < 0.001); RCA did not differ after adjustment. Likewise, the niFFR-like index was higher after MC in the LAD and LCX, with the largest shift in the LAD by 0.05 (HL: 0.04 [0.04–0.05]; r = 0.77; Holm-adjusted *p* < 0.001); the RCA change did not remain significant after Holm correction.

Although several paired comparisons remained statistically significant after Holm adjustment, the absolute MC-NMC differences were small for resting flow, particularly in global LV. Larger and more consistent numerical differences were observed for the LAD-sMBF, LAD-MFR, and LAD-niFFR-like index, as reflected by their paired differences, effect sizes, and HL estimates. Therefore, statistical significance was interpreted alongside absolute paired differences, effect sizes, and confidence intervals rather than *p*-values alone.

Patient-level directional analysis further clarified that MC did not simply increase or decrease the values (Supplementary Table S3). For rMBF, decreases after MC were most frequent in the LCX, occurring in 59.6% of patients, whereas the LAD showed a more balanced distribution after correction. In contrast, value increases after MC were more frequent for sMBF and MFR in the LV, LAD, and LCX. For the niFFR-like index, LAD showed the highest proportion of increase after MC, observed in 77.8% of patients. These findings demonstrate that the direction of MC-NMC differences varied across quantitative indices and vascular territories.

Despite the absolute shifts observed after MC, MC and NMC measurements remained strongly associated for most quantitative perfusion indices (Supplementary Table S4). Spearman analysis showed very high correlation for rMBF, sMBF, and MFR (rho = 0.93–0.99; all *p* < 0.001). The relative ranking of patients was largely preserved after MC. Passing–Bablock regression further showed that, for most MBF measures, intercepts (a) remained close to zero and slopes (b) were close to unity, supporting no constant or proportional bias between MC and NMC values. However, a more specific pattern emerged for MFR and niFFR. LV, LAD, and LCX showed evidence of constant bias, as their intercept confidence intervals (CI) excluded zero, whereas their slope CI remained inclusive of 1, reflecting no proportional bias. Among the niFFR-like index measures, LAD showed both constant and proportional bias, with an intercept of 0.2 (95% CI: 0.13–0.31) and a slope of 0.79 (95% CI: 0.69–0.89), while LCX showed constant bias only, as the intercept CI excluded zero, but the slope CI remained at the boundary of unity. Overall, these findings indicate that MC generally preserved the structural relationship of MBF and MFR measurements, whereas the niFFR-like index, particularly in the LAD, was more sensitive to correction-related changes.

**Figure 3:**
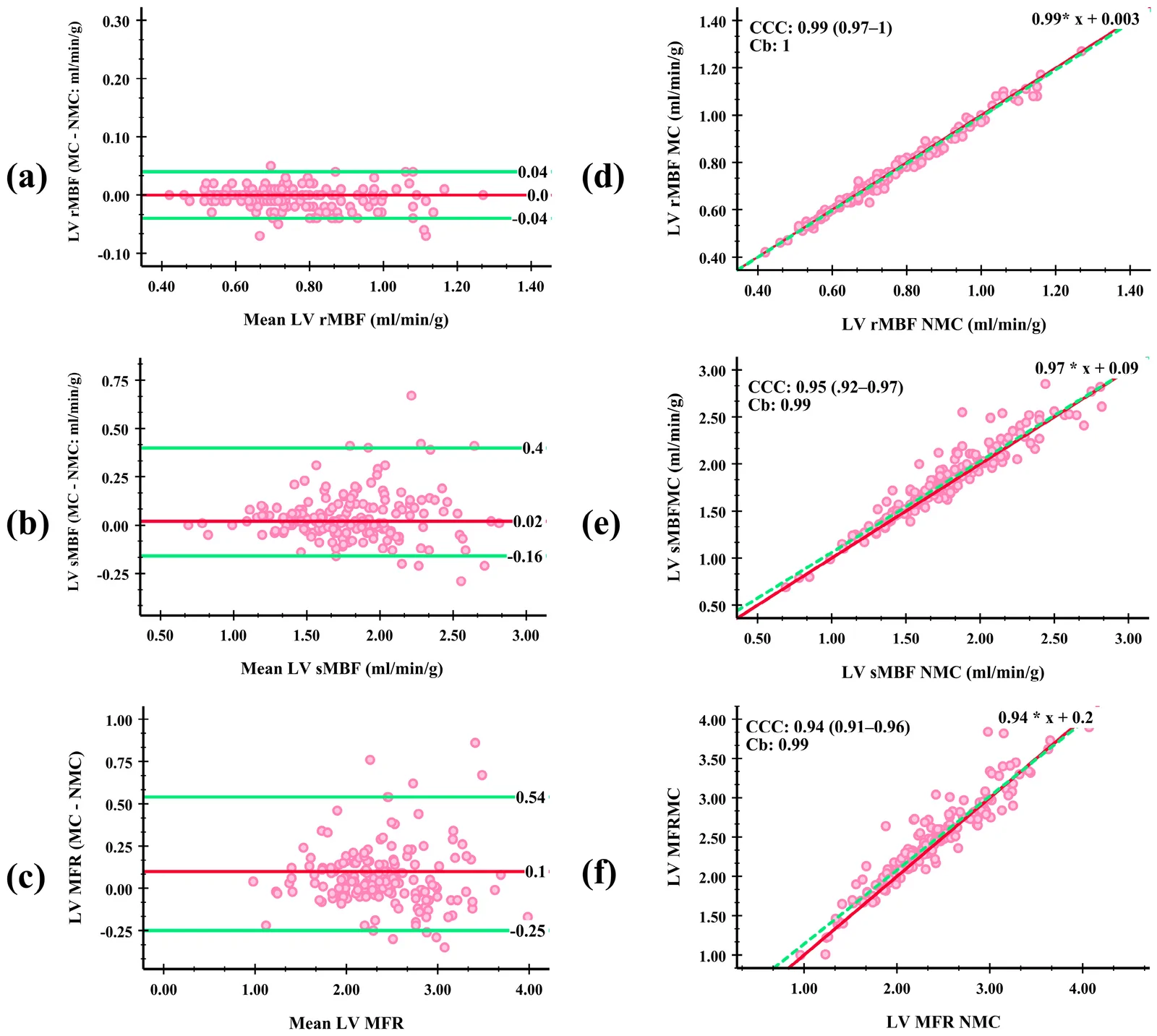
Agreement, precision, and accuracy between MC and NMC of global LV perfusion indices. Bland–Altman plots (left column) and Lin’s CCC analyses (right column) are shown for global LV: rest MBF (rMBF: (**a**,**d**)), stress MBF (sMBF: (**b**,**e**)), and myocardial flow reserve (MFR: **c**,**f**)). Footnotes (Figure 3): In Bland–Altman plots, the difference between MC and NMC values (MC − NMC) is plotted against their median. The solid red line indicates the median bias (median difference), and green lines show the 95% (2.5–97.5 percentile) limits of agreement (LoA). In Lin’s concordance plots, the solid red line represents the line of identity (45-degree), and the dashed line shows the fitted regression line; Lin’s CCC (with 95% CI) and the accuracy coefficient (Cb) are reported in each panel. MC, motion correction; NMC, non-motion correction; MBF, myocardial blood flow (rMBF; sMBF); MFR, myocardial flow reserve; LV, left ventricle.

Agreement and accuracy in absolute terms were evaluated using combined Bland–Altman and Lin’s CCC plots of global and regional territories. In global LV, rMBF showed virtually no systematic deviation, with a median difference of 0.0 mL/min/g and relatively narrow 95% limits of agreement (LoA: −0.04 to 0.04 mL/min/g). The data points cluster tightly around the bias line and the identity line, consistent with excellent concordance (CCC = 0.99) and accuracy relative to the identity line (red line) (Figure 3a,d). LV sMBF and MFR showed a small positive median difference (0.02 and 0.1) with broader LoA (sMBF: −0.16 to 0.4 mL/min/g; MFR: −0.25 to 0.54), consistent with greater dispersion after correction, particularly at higher values, where the spread of points becomes wider within LoA, and a few outliers are visible (Figure 3b,c,e,f). Nevertheless, concordance (CCC: 0.95 and 0.94) and accuracy (both Cb = 0.99) remained excellent, and most points still traced closely along the fitted line (Figure 3e,f).

Regional Bland–Altman and Lin’s CCC analyses showed small central MC-NMC differences for MBF and MFR across the LAD, LCX and RCA, although the dispersion of paired differences varied by indices and territory (Supplementary Figures S3–S5). For rMBF, median differences were 0.01 mL/min/g in the LAD and −0.01 m/min/g in both the LCX and RCA. Concordance was uniformly high (CCC: 0.9–0.99; Cb: 0.99–1), and the LAD demonstrated narrow LoA (−0.04 to 0.05 mL/min/g). Compared with rMBF, sMBF and MFR showed wider LoA, particularly in the LAD. Median differences in the LAD were 0.07 mL/min/g for sMBF and 0.12 for MFR, with LoA of −0.08 to 0.39 mL/min/g and −0.18 to 0.55, respectively; however, concordance remained high for both measures (CCC: 0.99; Cb: 0.98). In the LCX, median differences were 0.02 mL/min/g for sMBF and 0.11 for MFR, with CCC values of 0.94–0.95 and Cb of 0.99. In RCA, corresponding median differences were 0.03 mL/min/g and 0.07, with CCC values of 0.95 and 0.94, and Cb values of 1 and 0.99, respectively.

The niFFR-like index showed greater variability across specific territories (Supplementary Figures S3–S5). The LAD has the largest median difference (0.05; LoA: −0.03 to 0.19) and the lowest concordance (CCC: 0.68; Cb: 0.85). Although the LCX and RCA each had a median difference of 0.01, agreement was stronger in the RCA (LoA: −0.06 to 0.08; CCC: 0.9; Cb:0.99) than in the LCX (LoA: −0.04 to 0.14; CCC: 0.08; Cb: 0.97). Overall, these analyses indicate limited systematic MC-NMC differences, with modest positive shifts for LAD and LCX MFR and LAD niFFR-like index; agreement was strong for MBF and MFR but lower for LAD niFFR.

The combined Bland–Altman and Lin’s CCC plots indicate that MC and NMC measurements remained highly concordant for MBF and MFR, with the tightest agreement for rMBF and somewhat greater spread for sMBF and MFR, particularly at higher values, likely due to stress-induced physiological factors such as respiratory excursion, altered chest wall mechanics, and patient discomfort during the pharmacologic stress.

These quantitative shifts also translated into threshold-based classification for MFR (≥2) and niFFR-like index (>0.8). The 0.8 threshold was selected based on the established invasive FFR cut-off for hemodynamically significant stenosis, but is applied here exploratively, as this index has not been validated against invasive FFR. For MFR, the proportion of scans classified as ≥2 cut-off increased after MC across all territories, with statistical significance observed only in the LAD (increase from 69% to 77.2%; *p* = 0001) (Table 4). For the niFFR-like index, the largest reclassification occurred in the LAD, where the proportion of scans classified as >0.8 cut-off increased from 71.9% to 91.8% after correction (*p* < 0.001) (Table 4). Reclassification analysis revealed bidirectional changes for both metrics, with shifts from below-threshold to above-threshold ranging from 3.51% to 8.77% for MFR and 3.51% to 19.88% for the niFFR-like index, whereas in a few patients the shift was reversed from above-threshold to below-threshold (0.58% to 2.34% for MFR and 1.17% for the niFFR-like index) (Supplementary Table S5). The presence of shifts in both directions indicates that MC functioned as a bidirectional numerical adjustment rather than imposing a uniform shift, consistent with its motion behavior. Importantly, as this study lacks reference standards, these classifications should be interpreted as MC influence in PET-derived flow indices rather than as evidence of diagnostic improvement.

The motion magnitude analysis further supported the interpretation (Supplementary Table S6). In the >0–3 mm category, only three patients contributed rMBF, and the MC and NMC median difference was 0.01 mL/min/g. No sMBF, MFR, and niFFR-like index data were available in this group. In the >3–7 mm group, the difference between MC and NMC remained small, with little change in rMBF (−0.01 mL/min/g) and only slight upward shifts in sMBF and MFR (0.04 mL/min/g and 0.02 units). By contrast, in the >7 mm category, the observed differences were more pronounced, particularly for sMBF (0.05 mL/min/g) and MFR (0.11 units), whereas rMBF remained at 0.01 mL/min/g. Although the niFFR-like index showed no median change, its distribution rose slightly after correction. Overall, larger numerical differences were observed in higher motion categories and were most apparent for stress-derived indices.

Overall, the multifaceted analyses converged on a consistent pattern: motion during dynamic PET-MPI was more pronounced and heterogeneous during stress, and larger numerical differences between MC and NMC were observed under higher-motion conditions, particularly for stress-derived indices and in the LAD territory. Although corrected and non-corrected values remained strongly associated, they were not fully interchangeable. The numerical shifts were sufficient to alter threshold-based classification and the proportion of scans meeting predefined diagnostic cut-offs.

## 5. Discussion

In this PET MPI study, frame-by-frame motion analysis showed motion during both phases, more frequent and greater during stress, with the most displacement along the Y-axis. This motion affected downstream analyses: MC had a limited impact on rMBF but more on sMBF, MFR, and niFFR-like index, especially in the LAD territory. The impact of MC was consistent across various analyses, indicating structured, bidirectional changes in perfusion measures. The present study demonstrates, through multiple complementary statistical approaches, that MC is associated with measurable numerical differences in downstream quantitative indices and their threshold-based classification. These findings should be interpreted within a mechanistic framework, as the absence of an external gold standard precludes any claim of diagnostic validation or clinical superiority of the corrected values.

The 3D translational motion graph analysis showed measurable displacement along all axes, with notable frame-to-frame variability dominating different directions in both phases (Figure 2 and Table 2). Many PET studies have focused on Z-axis-dependent motion, which has been associated with increased MBF and MFR errors [4,5,6,7]. In contrast, the dominant stress displacement in our cohort was along the Y-axis rather than the Z-axis. This pattern may reflect changes in breathing pattern and diaphragmatic movement induced by a pharmacological stressor during dynamic stress, suggesting that the dominant axis of motion in dynamic PET-MPI may vary across studies [4,7].

The directionality and magnitude of motion were also relevant because regional perturbation was not expected to be uniform (Table 2, Supplementary Table S2). Several studies indicate that left–right (X), anterior–posterior (Y), and superior–inferior (Z) shifts alter both dynamic frame alignment and attenuation registration, with regional effects potentially involving the lateral, inferior, and anterior walls [4,5,6,7,19]. The vector pattern observed in our cohort was consistent with bidirectional numerical shifts observed after MC. For example, leftward displacement may alter lateral-wall counts through interaction with adjacent lung attenuation; posterior motion may influence lateral and inferior territories; anterior displacement may affect anterior or septal regions through right ventricular blood-pool spill-over, and inferior motion may alter the apparent activity of the inferior wall through diaphragmatic proximity, particularly during the stress phase [4,5,19]. These mechanisms should be viewed as plausible explanatory models rather than definitive proof, but they support the interpretation that the observed differences between MC and NMC were related to the specific motion pattern present in the datasets. In addition, the distribution of non-zero frame-wise motion magnitude suggests that the potential for quantitative differences increases with motion amplitude (Supplementary Tables S1 and S6).

The observed quantitative findings are internally consistent across the analytical approaches: rMBF showed little overall change, whereas sMBF and MFR showed more pronounced numerical difference after MC (Table 3; Supplementary Table S2). The stress-dominant pattern aligns with prior PET MC studies showing that correction influences stress-derived indices more than resting flow and can affect repeatability and reproducibility of dynamic PET flow quantification. Moreover, automated MC has been shown to alter MBF estimates and affect the predictive performance of stress MBF [10,11,12,13,19,20]. Our study extends this literature by showing that the numerical shift associated with MC was coherent across multiple statistical approaches and more evident with increasing motion severity (Supplementary Table S6). The robustness of these observed patterns was further supported by Hodges-Lehmann estimates, which confirmed the direction and magnitude of paired differences (e.g., LAD-sMBF: 0.06 [0.04–0.08] mL/min/g), and by the Bonferroni–Holm correction, which indicated that the majority of territorial findings remained significant after adjustment for multiple comparisons (Supplementary Table S2).

In regional correction, the pronounced correction was observed in the LAD, whereas the RCA was comparatively less affected in several analyses (Table 2 and Table 4; Supplementary Table S2 and Figures S3 and S5). This differs from prior PET studies, in which the RCA often showed prominent changes after correction [4,5,6,7,8,9,10,11,12,13,20]. Rather than representing a contraindication, this discrepancy is more likely related to differences in the actual direction, magnitude, and type of motion present in the dataset, as well as how that motion interacts with contour placement, blood-pool activity, and early-frame kinetics [1,4,5,21]. The marked anterior–posterior displacement observed under stress may have contributed to the more pronounced numerical shifts in the LAD territory, although a direct relationship cannot be established from the present data.

A central finding of the present study was that a strong association did not imply interchangeability. Spearman and PaBa regression analyses showed that patient ranking and structural relationship for MBF and MFR were largely preserved after MC, whereas Bland–Altman (BA) and Lin’s CCC analyses indicated that corrected and non-corrected values were not fully interchangeable in absolute terms, especially for stress-derived indices (Figure 3; Supplementary Table S4 and Figures S3–S5). The broad spread observed on the BA plots, despite most observations remaining within the LoA, suggests greater variability in paired differences rather than a complete absence of agreement. This likely reflects heterogeneity in the magnitude of numerical shifts across patients, depending on motion characteristics and acquisition phase. The broader spread at higher values may also indicate scale-related variability, as stress-derived indices are more susceptible to motion-related perturbation. This is consistent with studies showing that quantitative perfusion metrics are sensitive to technical and physiological factors. For example, SPECT MPI has been used to identify myocardial ischemia in long-COVID patients, with an abnormal summed stress score (SSS) serving as an independent predictor of ischemia [22]. Similarly, in pulmonary hypertension, increased right ventricular uptake on MPS has been shown to correlate with elevated pulmonary artery pressure [23]. These examples highlight the importance of technical quality in perfusion imaging, as small numerical shifts can influence patient classification. While MC and NMC values remained broadly associated, the observed numerical shifts were sufficient to influence threshold-based classifications (Table 4 and Supplementary Table S5), consistent with the practical PET reporting guidance emphasizing the importance of technical quality and acquisition stability [1,3,11,20].

Previous studies have reported association between PET-derived niFFR-like indices and invasive FFR [6,15,16]; however, in the present study, the niFFR-like index was not used as a surrogate reference standard. Rather, it was included as an additional lesion-oriented physiologic descriptor, which may be relevant in patients with focal coronary disease [15,16]. In our data, the moderate and significant correlation between MFR and niFFR suggests that these measures are related but not interchangeable, reflecting overlapping yet distinct aspects of coronary pathophysiology. MFR reflects integrated territory-level flow behavior across the epicardial, diffuse atherosclerosis, and microvascular function, whereas niFFR-like indices are closer to lesion-specific relative physiology [14,15,16]. Accordingly, the different behavior of MFR and the niFFR-like index in our analysis should be interpreted as complementary rather than conflicting. In this context, the lower concordance and greater scatter of the niFFR-like index, especially in the LAD, likely reflect the greater sensitivity of a lesion-oriented relative index to motion-related perturbation (Table 3 and Table 4; Supplementary Figure S3d,h and Table S5). Of note, the RCA niFFR-like index did not remain significant after Holm correction, further supporting the territorial heterogeneity (Supplementary Table S2). As the present study was not designed to validate this index against invasive FFR, these findings should be interpreted as exploratory and descriptive.

The reclassification results reinforce this interpretation. For both MFR and the niFFR-like index, threshold-based changes after MC were bidirectional, even though below-threshold to above-threshold (MFR ≥2 and niFFR-like index > 0.8) shifts were more frequent than the reverse in some territories, especially in LAD (Supplementary Table S5). These findings suggest that MC does not simply impose uniform upward shifts on perfusion values. Instead, they are consistent with the motion graph and directional analyses (Figure 2, Table 2). MC acts according to the direction and magnitude of motion and can therefore either increase or decrease downstream quantitative values depending on how the original dynamic frames were distorted [4,5,6,7,24]. The motion magnitude analysis supports the same interpretation, showing minimal effect at very low motion and progressively larger effects with increasing motion severity, particularly for stress-derived indices (Supplementary Table S6). This pattern is of potential clinical relevance, particularly within the borderline range, where technical perturbation may affect measurement interpretation and subsequent patient classification [1,3].

The observed numerical shifts associated with MC were consistent across complementary analyses of numerical shift, directional behavior, structural association, agreement, reclassification, and motion severity. The statistical methods used are well established, but their integrated application allowed for a coherent assessment of numerical change, bidirectionality, interchangeability, classification shift, and motion dependence. Overall, the observed patterns suggest that MC-related numerical shifts were motion-dependent, territorially heterogeneous, bidirectional, and sufficient enough to influence threshold-based classifications.

## 6. Conclusions

Dynamic PET-MPI showed more frequent, heterogeneous, and pronounced motion during stress than at rest, and these patterns were associated with bidirectional numerical shifts in perfusion indices after MC. MC consistently influenced quantitative datasets in a non-random manner, with stress-derived indices and lesion-oriented niFFR-like indices showing the largest observed difference across complementary analyses. Corrected and uncorrected MBF and MFR values were strongly associated but not interchangeable. The observed numerical shifts were sufficient to influence threshold-based classification and the proportion of scans meeting predefined cut-offs. These findings suggest MC is a relevant component of dynamic PET-MPI processing, with measurable consequences for quantitative interpretation.

## 7. Limitations and Recommendations

This study has several limitations. First, it was not designed to validate MC values against an external physiologic reference; therefore, the findings demonstrate the influence of MC on quantitative perfusion indices rather than the superiority of corrected measurement. Second, the dominant stress motion in this cohort occurred along the Y-axis, which differs from prior PET reports emphasizing superior–inferior motion, and the reasons for this difference cannot be fully determined from the present data. Third, the results reflect a single acquisition and processing workflow and may not generalize quantitatively across tracers, vendors, software platforms, or correction algorithms. Fourth, the motion vector analysis was performed at the frame level; although the Friedmann test was used to account for within-patient correlation, these findings should be interpreted with the nested structure of the data in mind. Fifth, the exclusion of discordant QA was a deliberate choice to avoid confounding and isolate the algorithmic influence of MC; however, this limits generalizability to discordant QA/failed QA rating cases, which future studies should address.

Future studies should evaluate MC in larger, prospective, and preferably multicenter PET-MPI with comparison across software platforms and correction approaches. Validation against invasive physiologic measurements and clinical outcomes is needed to determine the diagnostic and prognostic significance of motion-related reclassification. Further work should also clarify how motion direction influences territorial perfusion estimates and identify the motion severity threshold at which correction consistently affects quantitative interpretation.

## Figures and Tables

**Table 1:** Baseline characteristics of patients.

Variables	Median (IQR: P25–P75%)
Age (Years)	61 (54–69)
Height (Cm)	167 (160–172)
Weight (Kg)	71 (63–81)
Gender (Male)	95 (55.6%)
BMI	25.8 (23.9–28.5)
Age Groups (Years)	
≤50	26 (15.20%)
51–59	49 (28.66%)
≥60	96 (56.14%)
BMI Groups	
Underweight	1 (0.58%)
Healthy Weight	60 (35.09%)
Overweight	81 (47.37%)
Obese	29 (16.96%)
Menopausal—Females Only (*n* = 76)	56 (73.7%)
Elder	93 (54.4%)

Footnotes: Continuous variables are presented as medians (interquartile ranges), and categorical variables as counts and percentages. BMI, Body Mass Index.

**Table 2:** Frame-wise non-zero motion vectors for rest and stress axes (X, Y, and Z).

Variables Total Frames: 3591	Framewise Median Absolute Value (IQR: P25–P75%)	Framewise Direction (%)
Rest (Pixel Shift)		
X (Frame: 715)	0.75 (0.6–1.15)	Right (66.15%)
Y (Frame: 786)	0.7 (0.55–0.85)	Anterior (62.21%)
Z (Frame: 363)	0.65 (0.56–0.85)	Inferior (80.72%)
Stress (Pixel shift)		
X (Frame: 1341)	0.8 (0.65–1.05)	Left (73.6%)
Y (Frame: 1824)	1 (0.7–1.45)	Posterior (81.74%)
Z (Frame: 398)	0.7 (0.55–1.05)	Inferior (69.35%)

Footnotes: Values were based on non-zero frame-wise absolute pixel shift; non-zero frames were used for median absolute value; frame-wise direction percentage across all axes in rest and stress phases.

**Table 3:** Paired comparisons of quantitative perfusion indices before and after MC using the Wilcoxon signed rank test.

Variables	Median (IQR: P25–P75%)		Median Difference	Correction (%)	Z-Value	*p*-Value	Effect Size (r)
	MC	NMC					
Rest MBF (mL/min/g)							
LV	0.73 (0.64–0.85)	0.73 (0.65–0.87)	0	0	−3.2	0.001	0.3
LAD	0.73 (0.63–0.86)	0.72 (0.64–0.86)	0.01	1.39	0.87	0.38	0.08
LCX	0.73 (0.65–0.87)	0.74 (0.63–0.87)	−0.01	−1.35	−4.89	<0.001	0.41
RCA	0.75 (0.65–0.87)	0.76 (0.76–0.89)	−0.01	−1.32	−3.15	0.001	0.28
Stress MBF (ml/min/g)							
LV	1.77 (1.52–2.06)	1.75 (1.49–1.99)	0.02	1.14	3.68	<0.001	0.29
LAD	1.75 (1.48–2.02)	1.68 (1.4–1.99)	0.07	4.17	7.13	<0.001	0.56
LCX	1.79 (1.52–2.02)	1.77 (1.54–2.03)	0.02	1.13	2.61	0.009	0.2
RCA	1.86 (1.61–2.13)	1.83 (1.57–2.16)	0.03	1.64	−0.92	0.358	−0.07
MFR							
LV	2.44 (2.09–2.76)	2.34 (2.01–2.77)	0.1	4.28	4.4	<0.001	0.34
LAD	2.39 (2.01–2.72)	2.27 (1.92–2.67)	0.12	5.29	6.59	<0.001	0.51
LCX	2.45 (2.11–2.82)	2.34 (2.06–2.74)	0.11	4.7	4.68	<0.001	0.36
RCA	2.51 (2.11–2.82)	2.44 (2.08–2.8)	0.07	2.87	0.32	0.752	0.03
niFFR-Like Index							
LAD	0.91 (0.86–0.95)	0.86 (0.8–0.92)	0.05	5.82	9.48	<0.001	0.77
LCX	0.93 (0.89–0.97)	0.92 (0.86–0.96)	0.01	1.08	4.58	<0.001	0.38
RCA	0.93 (0.88–0.96)	0.92 (0.87–0.96)	0.01	1.08	2.46	0.013	0.21

Footnotes: Statistics are reported as the median (IQR). Median difference reflects the paired change between MC and NMC (MC − NMC). Percentage correction denotes the paired median relative change with respect to NMC values, calculated as {(MC − NMC)/NMC} × 100%. The Wilcoxon signed-rank test was used for paired comparison and two-sided *p*-values and z-values. Effect size (r) represents the magnitude of the paired difference. Quantitative perfusion indices: myocardial blood flow (MBF—mL/min/g), myocardial flow reserve (MFR), non-invasive fractional flow reserve (niFFR)-like index; LV, left ventricle; LAD, left anterior descending artery; LCX, left circumflex artery; RCA, right coronary artery.

**Table 4:** Normal scan rates in MC and NMC datasets with McNemar reclassification analysis and Spearman’s correlation coefficient between MFR and niFFR.

Quantitative Indices		Normal MFR Count (%)	McNemar (*p*-Value)	Normal niFFR-Like Index Count (%)	McNemar (*p*-Value)	Spearman Correlation MFR-niFFR	rho	*p*-Value
LV	MC	136 (79.5%)	0.12					
	NMC	130 (76%)						
LAD	MC	132 (77.2%)	0.001	157 (91.8%)	<0.001	MC—MC	0.42	<0.001
	NMC	118 (69%)		123 (71.9%)		NMC—NMC	0.38	<0.001
LCX	MC	140 (81.9%)	0.13	159 (93%)	0.04	MC—MC	0.28	<0.001
	NMC	135 (78.9%)		151 (88.3%)		NMC—NMC	0.18	0.02
RCA	MC	136 (79.5%)	0.75	160 (93.6%)	0.29	MC—MC	0.37	<0.001
	NMC	134 (78.4%)		156 (91.2%)		NMC—NMC	0.35	<0.001

Footnotes: Normal scans were defined as having above-threshold (MFR of ≥2 and a niFFR-like index >0.8). The counts and percentages of normal studies were reported separately for the MC and NMC datasets. Paired changes in classifications of normal versus abnormal (ischemic) findings between the MC and NMC groups were evaluated using McNemar’s test. Additionally, Spearman’s correlation coefficients were used to assess the association between MFR and niFFR-like index across both datasets.

## Data Availability

The datasets utilized and analyzed in this study can be obtained directly from the corresponding author upon request due to legal and ethical restrictions, ensuring transparency and facilitating further research.

## Supplementary Materials

The following supporting information can be downloaded at https://www.mdpi.com/article/10.3390/diagnostics16162610/s1: Figure S1: Flow diagram for patient selection and exclusion; Figure S2: Effects of motion correction on global LV and regional territories quantitative perfusion indices; Figure S3: Agreement, precision, and accuracy between MC and NMC of LAD perfusion indices; Figure S4: Agreement, precision, and accuracy between MC and NMC LCX perfusion indices; Figure S5: Agreement, precision, and accuracy between MC and NMC RCA perfusion indices; Table S1: Distribution of non-zero frame-wise motion magnitude across motion vector axes in both phases; Table S2: Hodges–Lehmann estimates with 95% confidence interval and Bonferroni–Holm-corrected *p*-values; Table S3: Proportion of patients with decreased, increased, or no change in quantitative perfusion indices after MC; Table S4: Association and bias analysis between before and after MC of quantitative perfusion indices; Table S5: Counts and percentages for MFR and niFFR classification changes following motion correction; Table S6: Global quantitative perfusion indices stratified by motion magnitude before and after MC in both phases (rest and stress).

## Abbreviations

CAD	Coronary Artery Disease
FFR/RFR/niFFR	Fractional Flow Reserve/Relative Flow Reserve/Non-invasive FFR
MAV	Media Absolute Value
MBF	Myocardial Blood Flow
MFR	Myocardial Flow Reserve
MC/NMC	Motion Correction/Non-MC
PET/CT	Positron Emission Tomography/Computed Tomography
TAC	Time–Activity Curve
