# Supplementary material for: The Effect of Motion Correction on Quantitative Perfusion Indices and Threshold-Based Interpretation in 13N-Ammonia Dynamic PET Myocardial Perfusion Imaging
Source: Diagnostics (Basel). 2026 Aug 17;16(16):2610. doi: 10.3390/diagnostics16162610 (PMC13511809; doi:10.3390/diagnostics16162610)
Supplement: Supplementary file 1 [file diagnostics-16-02610-s001.zip › diagnostics-4420013-supplementary.pdf]

Supplementary Table and Figures

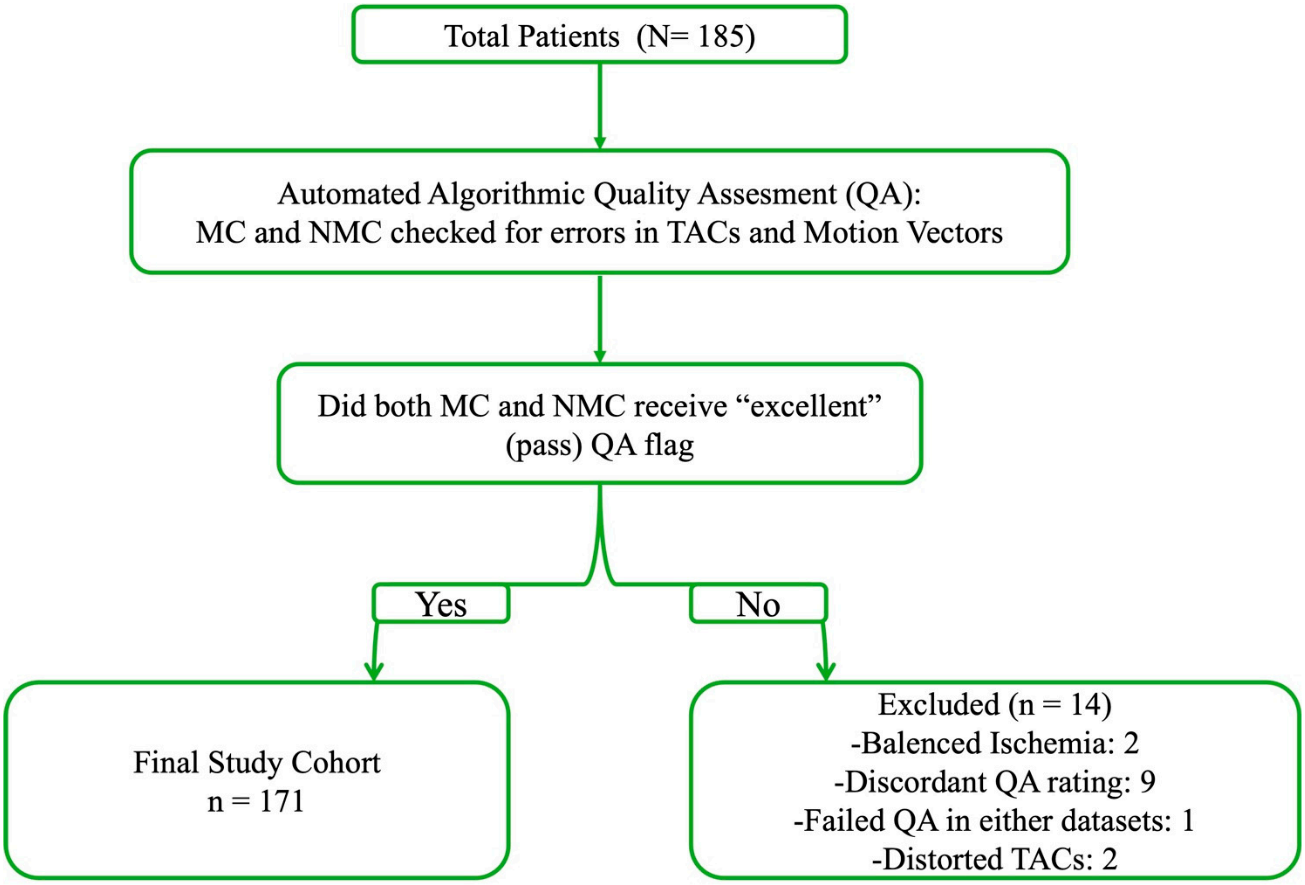

Supplementary Figure S1: Flow diagram for patient selection and exclusion. Of the 185 consecutive patients screened, 171 met the predefined automated QA criteria and were included in the final analysis. Fourteen patients were excluded due to balanced ischemia, discordant QA rating, failed QA in either dataset, or distorted TACs.

Supplementary Table S1. Distribution of non-zero frame-wise motion magnitude across motion vector axes in both phases.

Vector	Total Frame (3591)	>0–3 mm Count (%)	>3–7 mm Count (%)	>7 mm Count (%)
Rest				
X	715	263 (36.78%)	389 (54.41%)	63 (8.81%)
Y	786	392 (49.87%)	381 (48.47%)	13 (1.65%)
Z	363	200 (55.1%)	149 (41.05%)	14 (3.86%)
Stress				
X	1341	396 (29.53%)	874 (65.18%)	71 (5.29%)
Y	1824	418 (22.92%)	1075 (58.94%)	331 (18.15%)
Z	398	178 (44.72%)	182 (45.73%)	38 (9.55%)

Footnotes: Motion displacement was converted from pixel units to millimeters based on the reconstructed voxel size (one pixel equals 4.375mm, isotropic voxel dimension 4.375 × 4.375 × 4.375 mm). Non-zero motion categories are defined as >0–3mm, >3–7mm, and >7mm, and they are represented as count and categorical percentages.

Supplementary Table S2. Hodges–Lehmann estimates with 95% confidence interval and Bonferroni–Holm-corrected P-values.

Quantitative Indices	HL Estimates (95% CI)	Raw P-values	Holm-Adjusted P-values
Rest MBF			
LV	-–0.01(–0.01–0.01)	0.001	0.008
LAD	0.0 (0.0–0.01)	0.38	1
LCX	–0.01 [(–0.02)–(–0.01)]	<0.001	<0.001
RCA	–0.01 (–0.01–0.0)	0.001	0.009
Stress MBF			
LV	0.03 (0.02–0.05)	<0.001	0.002
LAD	0.06 (0.04–0.08)	<0.001	<0.001
LCX	0.02 (0.01–0.04)	0.009	0.046
RCA	–0.01 (–0.03–0.01)	0.358	1
MFR			
LV	0.05 (0.03–0.07)	<0.001	<0.001
LAD	0.08 (0.06–0.11)	<0.001	<0.001
LCX	0.06 (0.04–0.09)	<0.001	<0.001
RCA	0.01 (-0.02–0.03)	0.752	1
niFFR-Like Index			
LAD	0.04 (0.04–0.05)	<0.001	<0.001
LCX	0.01 (0.0–0.02)	<0.001	<0.001
RCA	0.01 (0.0–0.02)	0.013	0.052

Footnote: HL, Hodges–Lehmann; CI, confidence interval. The HL estimator is the pseudo-median of the paired differences (MC − NMC), with a 95% CI derived from the Wilcoxon signed-rank test. Holm correction was applied to global and territorial comparisons (n=15). Statistical significance after Holm correction (p<0.05).

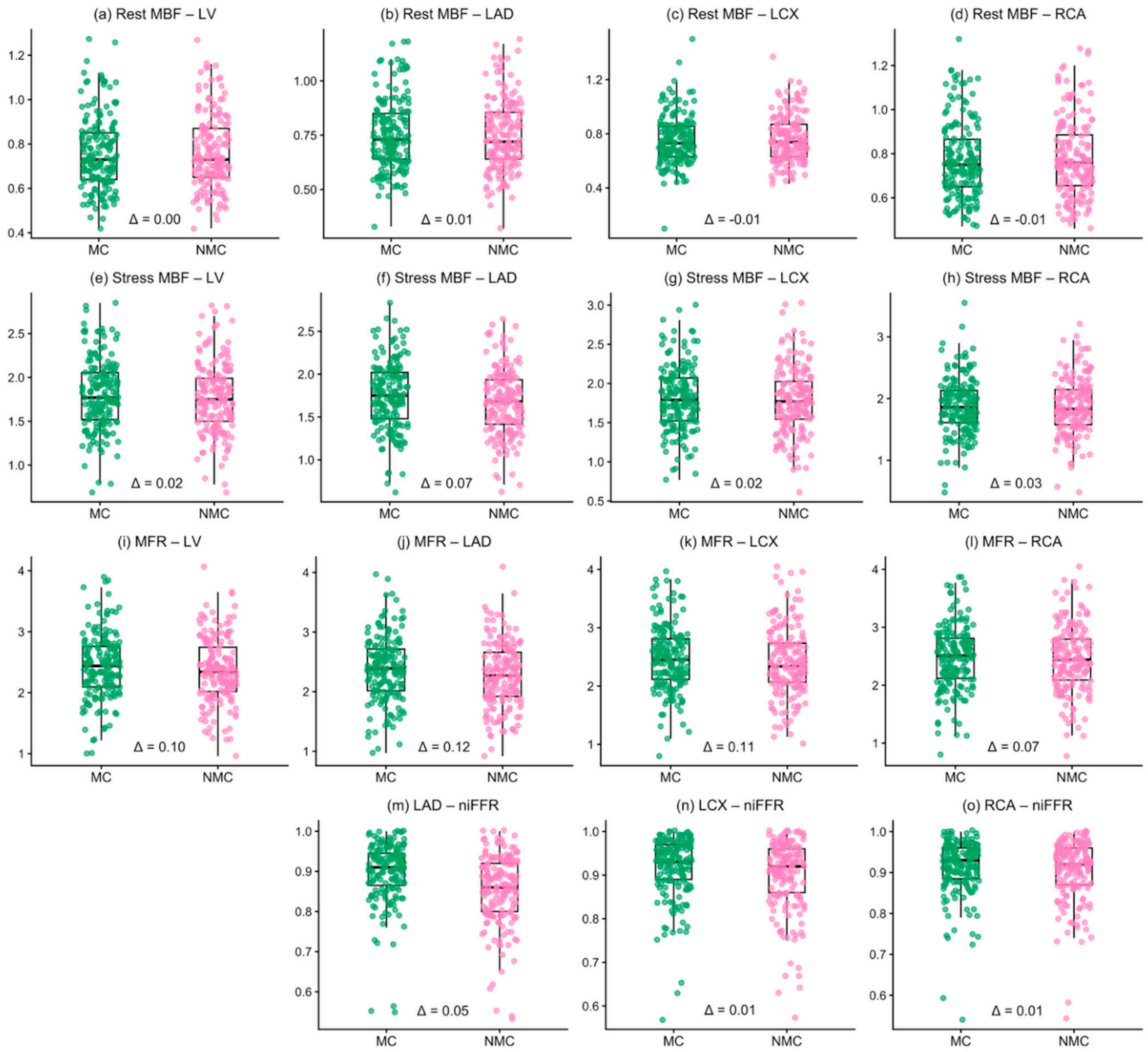

Supplementary Figure S2. Effects of motion correction on global LV and regional territories quantitative perfusion indices.

Footnotes: Box plots compare motion correction (MC, blue) and non-motion corrected (NMC, red) values for rest MBF (top row; a-d), stress MBF (second row; e-h), MFR, myocardial flow reserve (third row; i-l), and niFFR-like index, non-invasive FFR-like index (bottom row; m-o) in the global LV, LAD, LCX, and RCA territories. For each panel, boxes show the interquartile range, the horizontal line represents the median, the whiskers show the non-outlier range, and the points represent individual patients. Slightly above the X-axis, there was a median paired difference (MC − NMC) for the corresponding territory. MBF is expressed in ml/min/g; MFR and niFFR-like index are dimensionless.

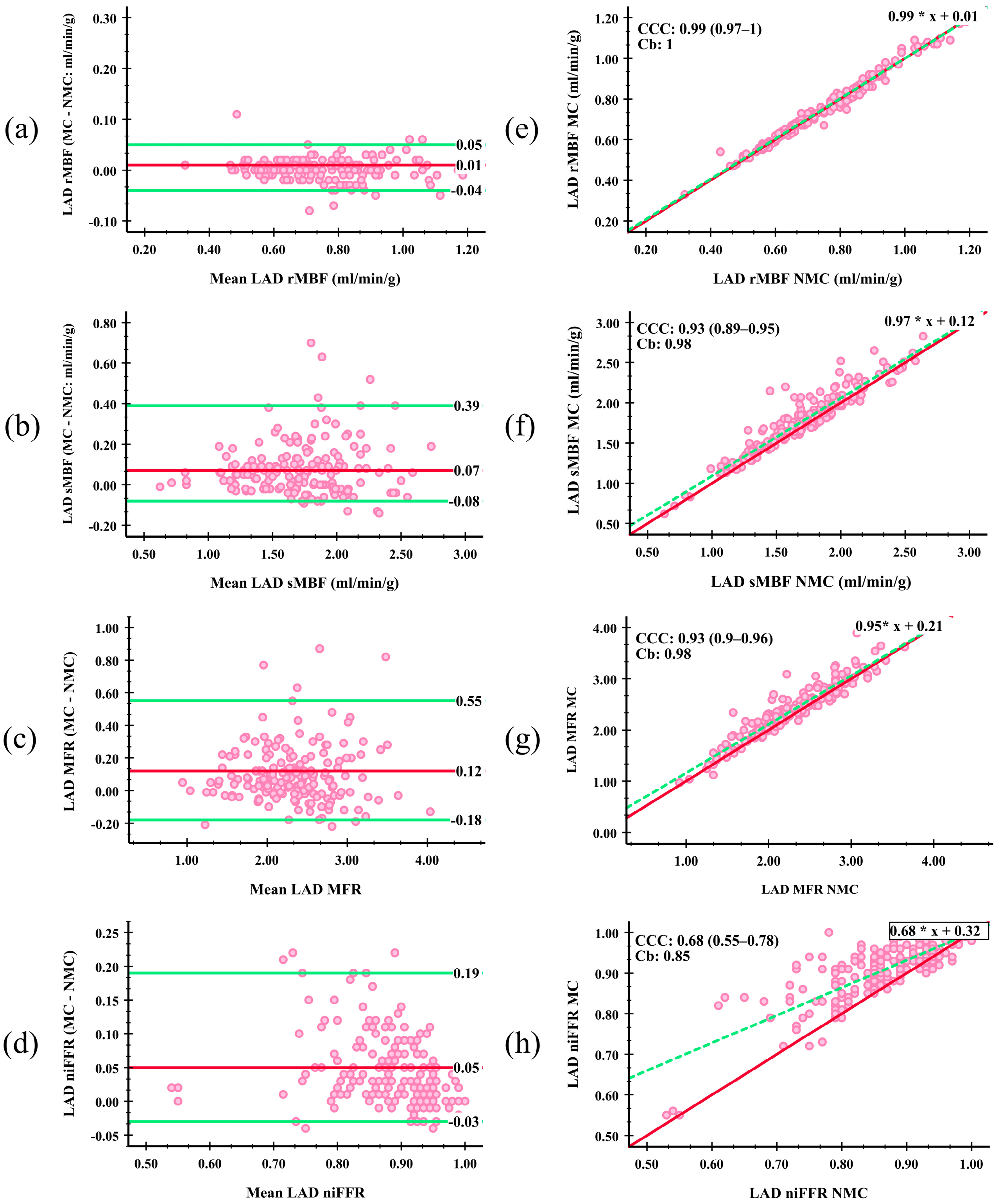

Supplementary Figure S3. Agreement, precision, and accuracy between MC and NMC of LAD perfusion indices. Bland–Altman plots (left column) and Lin's concordance correlation coefficient analyses (right column) are shown for LAD: rMBF (a, e), sMBF (b, f), MFR (c, g), and niFFR-like index (d, h).

Footnotes (S3-S5): In Bland–Altman plots, the difference between MC and NMC values (MC-NMC) is plotted against their median. The solid red line indicates the median bias, and green lines show the 95% (2.5-97.5 percentile) limits of agreement (LoA). In Lin's concordance plots, the solid red line represents the line of identity (45-degree), and the dashed line shows the fitted regression line; Lin's CCC (with 95% CI) and the accuracy coefficient (Cb) are reported

in each panel. MC, motion correction; NMC, non-motion correction; MBF, myocardial blood flow (rMBF, rest MBF; sMBF, stress MBF); MFR, myocardial flow reserve; niFFR-like index, non-invasive fractional flow reserve; LV, left ventricle; LAD, left anterior descending artery; LCX, left circumflex artery; RCA, right coronary artery.

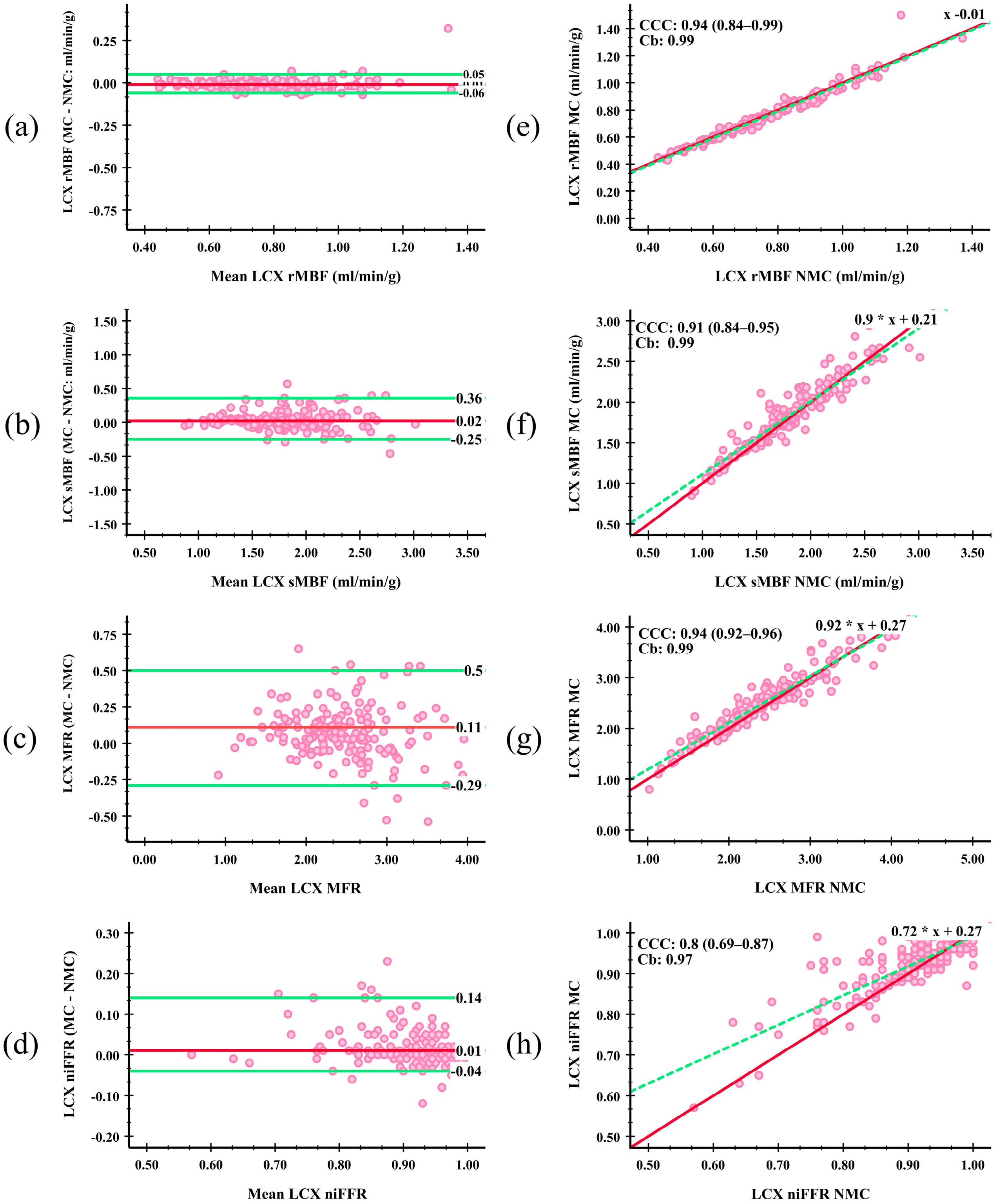

Supplementary Figure S4. Agreement, precision, and accuracy between MC and NMC LCX perfusion indices. Bland–Altman plots (left column) and Lin's concordance correlation coefficient analyses (right column) are shown for LCX: rMBF (a, e), sMBF (b, f), MFR (c, g), and niFFR-like index (d, h).

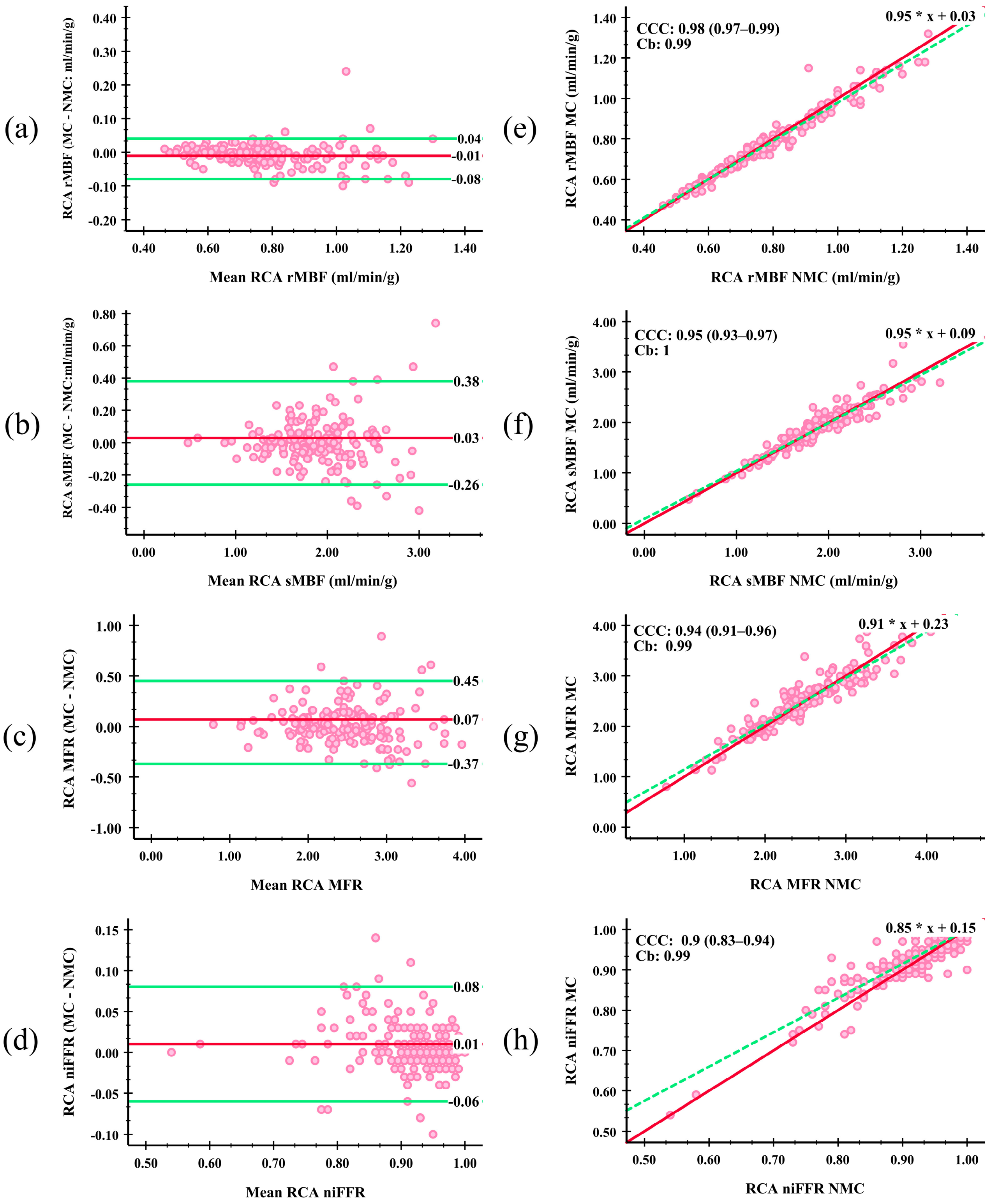

Supplementary Figure S5. Agreement, precision, and accuracy between MC and NMC RCA perfusion indices. Bland–Altman plots (left column) and Lin's concordance correlation coefficient analyses (right column) are shown for RCA: rMBF (a, e), sMBF (b, f), MFR (c, g), and niFFR-like index (d, h).

Supplementary Table S3. Proportion of patients with decreased, increased, or no change in quantitative perfusion indices after MC.

**Quantitative Indices**	**Decreased Count (%)**	**Increased Count (%)**	**No Change Count (%)**
Rest MBF			
LV	74 (43.3%)	41 (24%)	56 (32.7%)
LAD	56 (32.7)	67 (39.2%)	48 (28.1%)
LCX	102 (59.6%)	44 (25.7%)	25 (14.6%)
RCA	78 (45.6%)	54 (31.6%)	39 (22.8%)
Stress MBF			
LV	60 (35.1%)	102 (59.6%)	9 (5.3%)
LAD	49 (28.7%)	114 (66.7%)	8 (4.7%)
LCX	67 (39.2%)	97 (56.7%)	7 (4.1%)
RCA	87 (50.9%)	76 (44.4%)	8 (4.7%)
MFR			
LV	60 (35.1%)	104 (60.8%)	7 (4.1%)
LAD	52 (30.4%)	116 (67.8%)	3 (1.8%)
LCX	55 (32.2%)	115 (67.3%)	1 (0.6%)
RCA	85 (49.7%)	80 (46.8%)	6 (3.5%)
niFFR-Like Index			
LAD	19 (11.1%)	133 (77.8%)	19 (11.1%)
LCX	53 (31%)	92 (53.8%)	26 (15.2%)
RCA	66 (38.6%)	74 (43.3%)	31 (18.1%)

Footnotes: *The counts and percentages of decreased, increased, and no change were calculated by comparing MC values against NMC values on a paired basis, using NMC as the reference. Decreased counts are defined as MC < NMC, increased counts as MC > NMC, and equality as MC and NMC being identical for a given patient/territory.*

Supplementary Table S4. Association and bias analysis between before and after MC of quantitative perfusion indices

Quantitative Indices	Spearman (rho)	P-value	Intercept (a)	Intercept at 95% CI	Slope (b)	Slope at 95% CI
Rest MBF						
LV	0.99	<0.001	0	0.00–0.01	1	0.98–1
LAD	0.99	<0.001	0	0.00–0.00	1	1–1
LCX	0.98	<0.001	–0.01	(–0.01)–0.02	1	0.97–1
RCA	0.98	<0.001	0.02	0.00–0.04	0.97	0.95–1
Stress MBF						
LV	0.95	<0.001	0.02	(–0.05)–0.08	1	0.96–1.05
LAD	0.93	<0.001	0.06	(–0.02)–0.11	1	0.97–1.05
LCX	0.93	<0.001	0.02	(–0.05)–0.1	1	0.95–1.05
RCA	0.96	<0.001	0.02	(–0.05)–0.09	0.99	0.95–1.02
MFR						
LV	0.94	<0.001	0.11	0.02–0.21	0.97	0.93–1.01
LAD	0.95	<0.001	0.1	0.01–0.18	0.98	0.94–1.03
LCX	0.95	<0.001	0.14	0.03–0.25	0.97	0.92–1.01
RCA	0.94	<0.001	0.1	0.00–0.2	0.96	0.91–1
–						
LAD	0.75	<0.001	0.22	0.13–0.31	0.79	0.69–0.89
LCX	0.76	<0.001	0.09	0.01–0.18	0.91	0.82–1
RCA	0.85	<0.001	0.06	0.00–0.13	0.94	0.86–1

Footnote: Spearman's correlation coefficient measures the monotonic association between MC and NMC values. Passing–Bablok regression offers a non-parametric, robust assessment of both proportional and constant biases, providing the slope, intercept, and 95% confidence interval (from the 2.5th to 97.5th percentiles). It assumes no specific distribution and symmetric measurement errors. All analyses are performed on paired MC-NMC datasets, with significance determined at $p<0.05$. MC, motion correction; NMC, non-motion correction; MBF, myocardial blood flow (rMBF, rest MBF; sMBF, stress MBF); MFR, myocardial flow reserve; niFFR-like index, non-invasive fractional flow reserve; LV, left ventricle; LAD, left anterior descending artery; LCX, left circumflex artery; RCA, right coronary artery.

Supplementary Table S5. Counts and percentages for MFR and niFFR classification changes following motion correction.

**Quantitative Indices**		**MFR (≥2)**	**niFFR-Like Index (>0.8)**
LV	ABN - N	8 (4.68%)	
	N - ABN	2 (1.17%)	
LAD	ABN - N	15 (8.77%)	34 (19.88%)
	N - ABN	1 (0.58%)	0 (0%)
LCX	ABN - N	6 (3.51%)	10 (5.85%)
	N - ABN	1 (0.58%)	2 (1.17%)
RCA	ABN - N	6 (3.51%)	6 (3.51%)
	N - ABN	4 (2.34%)	2 (1.17%)

Footnotes: ABN-N= below-threshold to above-threshold shift after MC, N-ABN=above-threshold to below-threshold shift after MC. Values reflect reclassification using MFR: ≥2 and niFFR-like index: >0.8 cutoffs.

Supplementary Table S6. Global quantitative perfusion indices stratified by motion magnitude before and after MC in both phases (rest and stress).

**Motion Magnitude**	**Indices (n)**	**MC Median (IQR: P25% - P75%)**	**NMC Median (IQR: P25% - P75%)**	**Median Difference**
>0–3 mm	rMBF (3)	0.74 (0.72–0.76)	0.73 (0.72–0.75)	0.01
	sMBF (0)			
	MFR (0)			
	niFFR-Like Index (0)			
>3–7 mm	rMBF (105)	0.75 (0.72–0.79)	0.76 (0.72–0.8)	-0.01
	sMBF (53)	2.1 (1.81–2.38)	2.04 (1.78–2.34)	0.04
	MFR (36)	2.43 (2.25–2.75)	2.41 (2.15–2.78)	0.02
	niFFR-Like Index (36)	0.93 (0.89–0.96)	0.93 (0.87–0.95)	0
>7 mm	rMBF (63)	0.76 (0.72–0.81)	0.77 (0.72–0.82)	0.01
	sMBF (118)	1.97 (1.6–2.32)	1.92 (1.55–2.25)	0.05

MFR (135)	2.44 (2.09–2.77)	2.33 (1.99–2.69)	0.11
niFFR-Like Index (135)	0.92 (.89–0.96)	0.92 (0.87–0.96)	0

Footnote: Values are presented as median (IQR: P25% - P75%). Motion magnitude is defined based on the maximum frame-wise displacement per patient, calculated from the motion vectors (X, Y, Z) across all dynamic frames and converted from pixel shift to millimeters (1 pixel=4.375mm). Patients were categorized into three groups >0–3mm, >3–7mm, and >7 mm groups. rMBF=rest myocardial blood flow; sMBF= stress myocardial blood flow; MFR= myocardial flow reserve; niFFR-like index= non-invasive fractional flow reserve like index; NMC= non-motion correction; MC= motion correction.
